# First person – Vishwanath Varma

**DOI:** 10.1242/bio.047027

**Published:** 2019-08-15

## Abstract

First Person is a series of interviews with the first authors of a selection of papers published in Biology Open, helping early-career researchers promote themselves alongside their papers. Vishwanath Varma is first author on ‘Accuracy of fruit-fly eclosion rhythms evolves by strengthening circadian gating rather than developmental fine-tuning’, published in BiO. Vishwanath conducted the research described in this article while a PhD student in Prof. Vijay Kumar Sharma's lab at the Jawaharlal Nehru Centre for Advanced Scientific Research, India. He is now a postdoctoral fellow in the lab of Dr V. V. Binoy at the National Institute of Advanced Studies, India, investigating the links between animal behavior, physiology and life history, and how they adapt to changing ecological conditions.


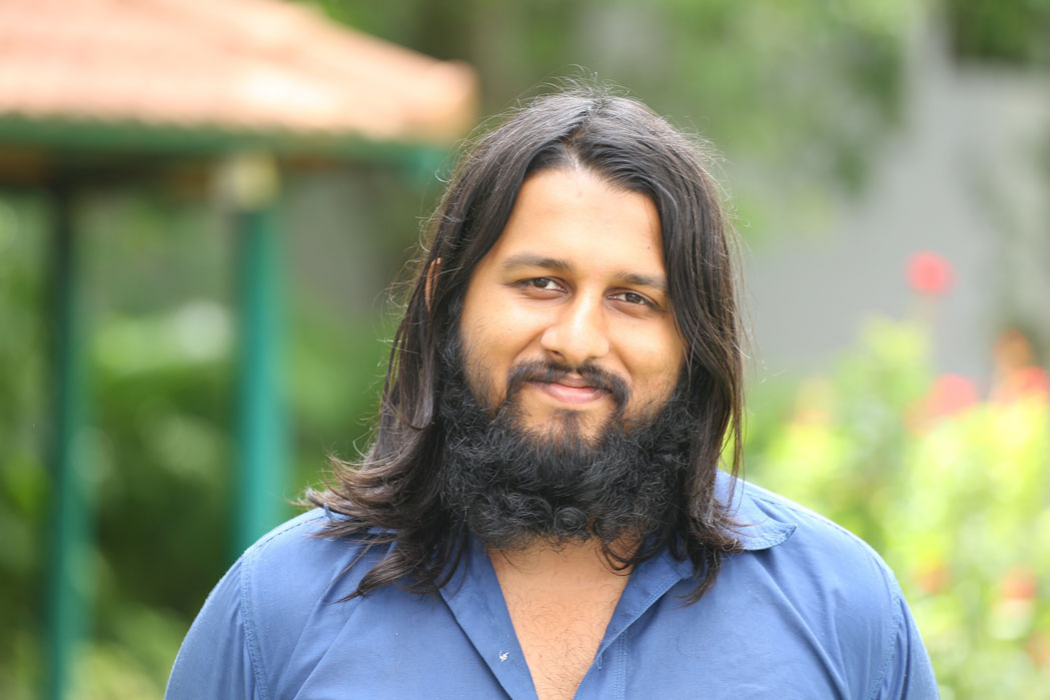


**Vishwanath Varma**

**What is your scientific background and the general focus of your lab?**

My background is in animal behavior, its ecology and evolution. During my PhD, I worked on the evolution of accurate biological rhythms in the fruit fly and investigated the factors thought to be important for optimal synchronization of the internal clock with the external environment. I also delved into questions on behavioral ecology such as the adaptation of the behavioral profile of flies to the continuously changing environmental variables of light and temperature and the effects of socio-sexual interactions on subsequent activity rhythms. My interests in social behavior and inter-individual variation in behavior led me to seek a lab working on personality and cognitive traits in fish, where I am currently a post-doctoral fellow. My current work includes behavioral flexibility of risk-taking, effects of social environment, and collective exploration and learning in fish.

**How would you explain the main findings of your paper to non-scientific family and friends?**

Most plants and animals have internal timers or biological clocks that help them keep track of changes in the environment. Such clocks allow animals to time their behavior to parts of the day best suited to express various activities such as finding food and mating. An important part of the life cycle of insects like flies is the emergence as adults from their pupal cases. This emergence is thought to be optimized to occur at times of minimal temperature and maximum humidity, though it was not clear how emergence can be effectively restricted to these parts of the day. My work shows that timing of emergence evolves to be more accurate mainly due to tighter gating of the final phase of development by the biological clock rather than eliminating general variation in development time among individuals or by becoming more sensitive to the external environment. In other words, the timing of emergence is more strictly controlled by the internal clock in flies that evolve greater accuracy even though earlier phases of development remain variable.

**What are the potential implications of these results for your field of research?**

While several studies have attempted to characterize variation in core clock genes, which determine the variability of the central clock, few studies focus on the output of the clock and its downstream effects on so-called peripheral oscillators. The results of my study suggest the availability of additive genetic variance or heritability of the control of behaviors by endogenous clocks that are amenable to selection pressures in the environment. Hence, polymorphisms in genes such as pigment-dispersing factor and prothoracicotrophic hormone, which mediate the influence of the central clock on eclosion, may be more likely targets of selection and more relevant to the evolution of accurate timing of eclosion. These may be extended to other behavioral and physiological variables under the control of the biological clock such as sleep–wake patterns or metabolic rhythms where focus may be shifted from genetic variation in the core clock to the messengers of the clock. The study also suggests that selection for accurate timing of particular behaviors does not necessarily require that the behaviors themselves become less variable. Instead, it is the control of the clock upon them that results in reduced variation in their timing.

**What has surprised you the most while conducting your research?**

“Even biological systems as simple as fly populations can present a strong case for preserving diversity in the world.”

The persistence of individual variation despite selection against it. One would imagine that strong selection for a trait would wipe out genetic variation for the trait. However, it appears that several processes, including trade-offs between fitness traits and frequency-dependent selection, can sustain the coexistence of multiple behavioral and life-history strategies in the population. Indeed, heterogeneity is known to be an important factor in resilience of complex ecological and socio-economic systems. Thus, even biological systems as simple as fly populations can present a strong case for preserving diversity in the world.

**Figure:**
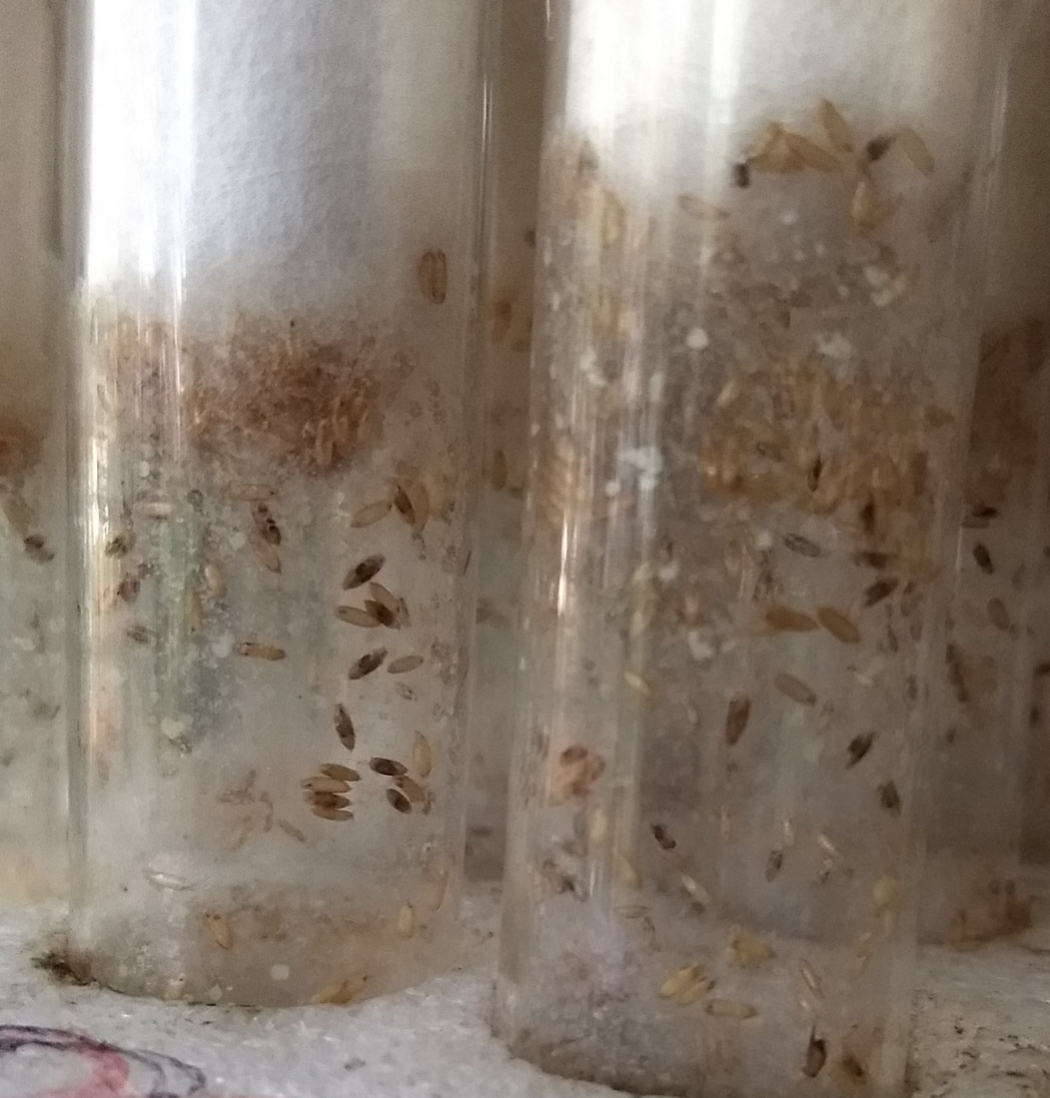
**Apparatus used for simultaneously assaying the timing of emergence of adult flies from nearly 10,000 pupae.**

**What, in your opinion, are some of the greatest achievements in your field and how has this influenced your research?**

While the explosion of the field of molecular chronobiology (for which the pioneers of this field were awarded the Nobel Prize in Medicine in 2017) has definitely revealed remarkable insights into the underlying structure and function of core circadian clocks, some of the advances in the plasticity of timing has been equally disruptive, in my opinion. While animals have long been classified as diurnal or nocturnal, some studies have shown that manipulating environmental conditions such as temperature and food availability can result in temporal niche switching in rodents. Moreover, recent work on the determinants of diurnality and nocturnality has shown that neuronal subgroups downstream of the core clock may be more important to the timing of behavior than the core clock itself. My work on the evolution of accurate timing of emergence also reveals a similar theme of clock control of behavior being more important than the clock or behavior themselves. This has also inspired my interest in the factors affecting behavioral flexibility in timing in fishes which are known to be less rigid in their sleep–wake patterns.

**What changes do you think could improve the professional lives of early-career scientists?**

Advances in science often proceed at a breakneck speed and can leave behind PhD researchers pursuing a single research question for over 5–6 years. While specialization and immersion in a particular field is essential to an in-depth understanding of a research problem, diversification and updating of skills required in the next phase of one's career is also important since there are few labs worldwide working on closely related questions. Promoting greater awareness of recent trends in the relevant fields could be useful in this regard. Another improvement could be the introduction of career counseling for early-career researchers, since guides may not always have the time to assist in proposal writing or advising on the job opportunities to target. A clearer understanding of what grant committees and employers look for in a candidate could help budding scientists prepare accordingly.

**What's next for you?**

Currently, I am pursuing research on behavioral and cognitive flexibility in an endangered freshwater fish. I am looking to expand into socio-ecological interactions and collective behavior in changing habitats that present new risks and opportunities due to human interventions. In the future, I would like to work on how individuals with different personalities (or natural behavioral tendencies) carve out their social niche and how communities might benefit from heterogeneity in constituent members while dealing with complex environments.
